# Association among depression, cognitive impairment and executive dysfunction after stroke

**DOI:** 10.1590/S1980-57642012DN06030007

**Published:** 2012

**Authors:** Luisa Terroni, Matildes F.M. Sobreiro, Adriana B. Conforto, Carla C. Adda, Valeri D. Guajardo, Mara Cristina S. de Lucia, Renério Fráguas

**Affiliations:** 1 Consultation-Liaison Psychiatry Group, Department and Institute of Psychiatry, Clinics Hospital, Medical School, University of São Paulo, São Paulo SP, Brazil.; 2 Neurology Department, Clinics Hospital, Medical School, University of São Paulo, Teaching and Research Israeli Institute, Albert Einstein Israeli Hospital, São Paulo SP, Brazil.; 3 Division of Psychology, Clinics Hospital, Medical School, University of São Paulo, São Paulo SP, Brazil.

**Keywords:** stroke, depression, cognition impairment, cognition, executive function, dysfunction, neuropsychological assessment

## Abstract

The relationship between depression and cognitive impairment, frequent after stroke, is complex and has not been sufficiently elucidated.

**Objective:**

To review the relationship between post-stroke depression and cognitive impairment.

**Methods:**

We performed a PubMed database search spanning the last ten years, using the terms post-stroke depression, cognitive dysfunction, cognitive impairment and neuropsychological tests. Our target studies were original quantitative studies that investigated the relationship between post-stroke depression (PSD) and cognitive impairment in stroke patients. Articles published in English, Spanish, Italian and Portuguese were considered. Selection criteria were the use of neuropsychological tests to assess cognitive function, and of either instruments to diagnose major depression, or scales to assess depressive symptoms, within the first three months after stroke.

**Results:**

Six original quantitative studies fulfilled the criteria. The prevalence of PSD within the first three months after stroke ranged from 22% to 31%. Incidence ranged from 25% to 27% and was evaluated in only two studies. PSD was associated with increased cognitive impairment. Cognitive impairment was reported in 35.2% to 87% of the patients. Post-stroke cognitive deficits were reported mostly in executive function, memory, language, and speed of processing.

**Conclusion:**

Executive dysfunction and depression occur in stroke survivors, are frequently coexistent, and also associated with worse stroke prognosis. Healthcare professionals need to address and provide adequate treatment for depression and executive dysfunctions in stroke patients early in the first three months after stroke. Future studies should evaluate the efficacy of programs evaluating the early detection and treatment of PSD and executive dysfunction in stroke survivors.

## Abstract (pt)

A depressão e o déficit cognitivo são frequentes após o acidente vascular cerebral. A relação entre as duas condições é complexa e não tem sido suficientemente elucidada.

**Objetivo:**

Fazer uma revisão da relação entre a depressão pós-AVC e prejuízo cognitivo.

**Métodos:**

Foi realizada uma pesquisa no banco de dados PubMed nos últimos dez anos. Nosso foco foi estudos quantitativos originais que investigaram a relação entre depressão e comprometimento cognitivo em pacientes com AVC. Os termos depressão, disfunção cognitiva, comprometimento cognitivo e testes neuropsicológicos foram usados para pesquisa. Foram considerados artigos publicados em Inglês, Espanhol, Italiano e Português. Os critérios de seleção foram o uso de testes neuropsicológicos para avaliar a função cognitiva com também o uso de instrumentos para diagnosticar a depressão maior ou escalas para avaliar sintomas depressivos nos primeiros três meses após o AVC.

**Resultados:**

Seis estudos quantitativos originais preencheram os critérios de seleção. A prevalência de depressão pós-AVC nos primeiros três meses após o AVC variou de 22% a 31%. A incidência de depressão pós-AVC variou de 25% a 27% e foi avaliada apenas em dois estudos. A depressão pós-AVC esteve associada com comprometimento cognitivo mais acentuado. Disfunção cognitiva pós-AVC foi relatada em 35,2% a 87% dos pacientes. Déficits cognitivos pós-AVC foram encontrados principalmente em função executiva, memória, linguagem e velocidade de processamento.

**Conclusão:**

A disfunção executiva e a depressão ocorrem em pacientes de AVC, sendo frequentemente concomitantes e associadas a um pior prognóstico. Os profissionais de saúde devem diagnosticar e fornecer um tratamento adequado para a depressão e disfunção executiva nos primeiros três meses após o AVC. Mais estudos devem avaliar a efetividade de programas de detecção precoce e tratamento da depressão e disfunção executiva pós-AVC.

## INTRODUCTION

Depression is the most common psychiatric complication of stroke with significant prevalence and incidence rates. The reported prevalence of major depression within three months after stroke ranges from 22% to 31%.^1-5^ The incidence rates of major depression after stroke (PSD) are little explored in the literature because most studies have included patients with past history of depression.^2,6-9^ Three studies have investigated the incidence of PSD after a stroke, Gainotti et al., reported an incidence of 27% 2-4 months after the stroke;^10^ Spalletta et al., found a rate of 25% between 3 weeks and 3 months after stroke,^3^ and Terroni et al. reported an incidence of 28.8% 3-4 months after first stroke.^11^ Besides its high frequency, depression has a negative impact in the post-stroke phase. PSD, or even depressive symptoms after stroke, have been associated with increased mortality, greater impairment in physical functioning and language, longer hospitalization, reduced quality of life and cognitive impairments.^12-14^ Studies have supported that PSD has underlying biological and psychosocial etiologic factors such as cognitive impairment, female sex, hypercortisolism, poor social network, living alone and previous depression.^2,6,9,15-17^

Studies on cognitive impairment after stroke have reported rates ranging from 35.2% to 87%.^18-20^ Similarly to PSD, cognitive impairment in stroke has been associated with negative outcomes such as reduced functional recovery and increased risk of mortality.^18,21-23^ Moreover, cognitive impairment may represent a sign of changes in evolution to degenerative diseases.^24^ It has been reported that 55% of patients have impairment in at least one cognitive domain,^25^ and frontal network syndromes have been found in 51% of subjects.^20^ Notably, executive dysfunction has been reported to range from 18% to 40%.^26-29^

Considering the high prevalence of depression and cognitive impairment, particularly cognitive dysfunction after stroke, their negative impact on prognosis and mortality, and the fact they are comorbid, render it important to study their relationship. Thus, the aim of this study was to review the literature on the association between PSD and cognitive impairment.

## METHODS

Key words used for this review were "post-stroke depression", "cognitive impairment", "cognitive dysfunction", and "neuropsychological tests". All studies in PubMed published between 2002 and 2012 retrieved based on these key words by the electronic search were reviewed for inclusion. Reference lists of studies included in the present review were also searched in order to identify additional citations. Studies in English, Portuguese, Spanish and Italian languages were included. Our target studies were original quantitative studies investigating the relationship between post-stroke depression and cognitive impairment. Inclusion criteria for the present review were as follows:

[A] original quantitative study;
[B] studies examining cognitive function using neuropsychological tests, not only the Mini-Mental State Examination (MMSE);
[C] the presence/absence of depression determined using a standardized diagnostic interview procedure or valid rating scale for depression;
[D] study participants limited to stroke patients; and
[E] evaluation performed within the first three months of stroke.

Review articles, case reports and unpublished data were excluded. The search was limited to studies involving adult human participants.

## RESULTS

The electronic search yielded forty potential studies for inclusion. Selection of studies involved initial screening of titles and abstracts to determine whether each study might meet the inclusion criteria stated above. A total of 37 studies were excluded. The reasons for exclusion were:

[A] time period of evaluation longer than 3 months, N=5;
[B] Chinese, Romanian, German, Russian, French languages, N=7;
[C] reviews, N=5;
[D] not depression and cognitive function investigation study, case report study, randomized clinical trial or no use of neuropsychological tests for cognitive evaluations, N=19; and
[E] study participants with transient ischemic attack, N=1. Based on the stated inclusion criteria, three original quantitative studies were identified for the present review.

After manual search, a further three studies were included that also met the inclusion criteria for the present review despite having been published before the previously established publication search period. The reasons behind the decision to include them were:

[1] the study performed by Bolla-Wilson et al.^30^ was the first to use neuropsychological tests to assess cognitive function;
[2] the studies of Pohjasvaara et al.^27^ and Vataja et al.^31^ included partially the same cohort of patients but given the different aims and methods employed by the two studies they were both included.

The four original quantitative studies identified for the present review, as well as the two earlier studies that used neuropsychological tests within three months of stroke,^27,31-34^ are listed in Table 1.

**Table 1:** Studies investigating the association between depression and cognitive function within three months of stroke.

Study	Characteristics of patients	Neuropsychological tests and type of cognitive impairment associated with depression	Depression evaluation
Bolla-Wilson et al. (1989)^30^	< 60 days, N=53Cross-sectional	Tests: Boston Naming Test,Reciting of Months Forward, Repetition of Words and Phrases,Verbal Fluency,Comprehension of Complex Ideational Material, Logical memory - immediate and delayed recall, Recurrent Words, Digit Span, Visual Reproduction, Recurrent words, Logical memory, Rey Auditory Verbal Learning test, Visual Reproduction, Block Design, Clock Drawing Test, Hooper Visual Organization Test, Verbal fluency for a single letter and animals, Alternating Fingers, Luria Motor Sequences.Findings: impairment in orientation, language, executive/motor function.	DSM-III45% of patients with LH lesion had major depression
Kauhanen et al. (1999)^32^	1-7 days, N=1063 months, N=101First strokeFollow-up study	Tests: 5 subtests of the Wechsler Adult Intelligence Scale-Revised, subtests of the Wechsler Memory Scale, serial learning and interference tasks, and visual recognition memory task, Trail-Making Test A, Verbal Fluency, copying tasks and modified clock hand task.Findings: visual and verbal memory, nonverbal problem solving, attention and psychomotor speed.	DSM III-R53% depressed at 3 months9% major depression44% minor depression
Pohjasvaara et al. (2002)^27^*	3-4 monthsN=256 patientsN=37 controlsCross-sectional cohort study	Tests: Wisconsin Card Sorting Test, Trail Making test, Stroop test, Verbal Fluency.Finding: executive dysfunction.	BDI
Nys et al. (2005)^33^*	3 weeks N=126 patients with first strokeN=87 controlsCross-sectional study	Tests: Raven Advanced Progressive Matrices; Rey Auditory Verbal Learning Test and Rey-Osterrieth Complex Figure-Delay; Brixton Spatial Anticipation Test; Test of Everyday Attention; Judgment of Line Orientation; Test of Facial Recognition; Digit Span; Token test; Boston Naming Test.Findings: domain of memory, visual perception and language.	MADRS40% mild depressive symptoms12% moderate/severe depressive symptoms
Vataja et al. (2005)^31^*^#^	3 monthsN=158 patientsN=28 controlsCross-sectionalCohort study	Tests: Nelson's version of the Wisconsin Card Sorting Test, Stroop test, Trail-Making A and B tests, Verbal fluency.Findings: 33.5% of patients with executive dysfunction and 13.3% with depression-dysexecutive syndrome.	SCANMADRS39.2% depressed patients
Barker-Collo (2007)^34^	3 monthsN=73 patientsFirst stroke Cross-sectional study	Tests: CVLT-II, VPA, IVA-CPT, Digit and spatial spans, Victoria Stroop.Findings: reduced cognitive speed, poorer verbal memory, and increased impact of interference (Stroop ratio).	BDI22.8% moderate or severe depression

* Findings in the electronic search of PubMed;

# study sample was part of study sample of Pohjasvaara et al. (2002) but not the same patients. In study by Vataja et al. (2005), authors included only those patients who did not fulfill the DSM-III criteria for dementia and had psychiatric examination; LH: left hemisphere; RH: right hemisphere; CVLT-II: California Verbal Learning Test-II; VPA:Visual Paired Associates; IVA-CPT: Integrated Visual Auditory Continuous Performance Test; MADRS: Montgomery Asberg Depression Rating Scale; BDI: Beck Depression Inventory; SCAN: Schedules for Clinical Assessment in Neuropsychiatry; DSM III- R: Diagnostic and Statistical Manual of Mental Disorders, Revised, 3^rd^ edition.

**Association between post-stroke depression and cognitive impairment.** Two early studies investigated the relationship between depression and cognitive domains (Table 1). The first found that patients with left hemisphere lesions and major depression performed significantly worse compared with non-depressed patients in four of nine cognitive domains.^30^ The authors concluded that post-stroke major depression appeared to produce a decline in cognitive performance dependent on the laterality of the lesion. Along the same lines, Kauhanen et al.^32^ examined 106 patients with first stroke at 1 to 7 days, 3 months and 12 months, after stroke using an extensive battery of neuropsychological tests. The presence of major depression after stroke was associated with impaired visual and verbal memory, nonverbal problem solving, attention and psychomotor speed at 12 months after stroke.

Recently, the association between depression symptomatology and cognitive impairment was investigated in 126 patients with first stroke at 3 weeks post-stroke.^33^ Of the included patients, 40% demonstrated mild, and 12% moderate to severe, depressive symptoms. Even after adjusting for lesion size, the neuropsychological profile of patients with moderate to severe depressive symptoms primarily showed compromise of memory, visual perception and language. A significant relationship was found in this study between severity of depressive symptoms and cognitive impairment. Patients with severe depressive symptoms had three times more cognitive impairment compared to patients with mild depressive symptoms. Interestingly, the authors suggested that depressive symptoms early after stroke are, at least in part, a reactive phenomenon secondary to severe cognitive deficits. Along the same line, Barker-Collo found that 74.6% of variance in depression after stroke was explained by significant relationships between reduced cognitive speed, poorer verbal memory, and increased impact of interference (Stroop ratio) besides other factors. Cognitive performance explained the greatest proportion of variance, 51.3%, in depression. The author concluded that "The findings suggest that cognition and mood are linked over and above physical independence and that both should be addressed as part of the rehabilitative process".^34^

Pohjasvaara et al. initially published a study that included 256 patients, aged 55-85 years, evaluating patients 3 and 4 months after stroke. The authors reported that 40.6% (N=104) of patients had executive dysfunction. Also, executive dysfunction was shown to be associated with depression symptoms.^27^ Recently, Vataja et al. described the depression-executive dysfunction syndrome after stroke. These authors reported that patients with depression-executive dysfunction had more severe depressive symptoms. Three months after ischemic stroke, brain infarcts affecting the frontal-subcortical circuit structures were significantly more frequent in patients with depression-executive dysfunction than in post-stroke subjects without depression-executive dysfunction.^31^

## DISCUSSION

The early studies assessing cognitive function using neuropsychological tests in the first three months after stroke reported associations between specific neuropsychological profiles and depression. Impairment in orientation, language, executive/motor function, memory, attention and psychomotor speed have been associated with depression.^30,32^ More recently, executive function impairment has been associated with depression after stroke and some authors have considered the existence of depression-dysexecutive syndrome within three to four months of stroke.^27,31^ Depression-executive dysfunction syndrome has been defined as late-onset depression associated with executive dysfunction.^35^ The executive function refers to a set of complex functions some of which include initiation / volition, planning, survey hypotheses, flexibility of thought, decision making, self-regulation, judgment, use of feedback and self-perception, amongst others.

Other studies have described the association between severity of PSD and magnitude of cognitive deficits.^32^ Cognitive impairment was shown to be three times more frequent in patients with more severe depression compared to those with mild symptoms.^33^ In addition, depression was more intense in patients with depression-executive dysfunction than in those without.^31^

Some studies have found that cognitive impairment after stroke is an important predictor of long-term depressive symptoms.^34^ The prognostic value of cognition suggests a reactive component in the development or continuation of long-term depressive symptoms.^36^ On this matter, some authors have proposed that depressive symptoms are, at least in part, a secondary reaction to cognitive deficits in post-stroke patients^32,37^ although the subject remains controversial. Studies evaluating the effects of treatment of depression on cognitive function have yielded some contributions to this question.

Treatment of PSD has been associated with cognitive improvement suggesting that cognitive impairment is a symptom or consequence of depression.^38-42^ For instance, Narushima et al.^43^ reported that in patients with early and sustained remission of depression there was a rapid improvement in cognitive function and this improvement persisted over a 2-year period. Furthermore, cognitive function remained unchanged in non-depressed patients over a 2-year follow-up. Even when only mood was considered, patients with post-stroke major depression whose mood improved at follow-up had significantly greater recovery in cognitive function than patients whose mood did not improve.^44^ Amelioration of depressive symptoms and cognitive impairment was described after anodal stimulation to the left dorsolateral prefrontal cortex (DLPFC).^40^ In this case report, it was not possible to conclude whether the improvement in cognitive function was secondary to depression improvement or whether the effects were independent of each other. On the other hand, improvement in global cognitive function, specifically in verbal and visual memory functions, was found to be independent of effects on depression after treatment with escitalopram in a randomized trial.^45^ Therefore, we conclude that the beneficial effect of treatment of depression in the process of cognitive post-stroke recovery should be further investigated by prospective studies and clinical trials.

Although treatment of depression is effective and can be associated with cognitive recovery, underdiagnosis of PSD and cognitive disorders after stroke has been reported. Depression may be diagnosed in only 20% to 50% of depressed stroke patients.^46^ Cognitive impairments are often inaccurately diagnosed. After applying a screening battery of tests for cognitive evaluation, thirty-five percent of patients was found to have more cognitive impairments than previously diagnosed by the assisting treatment team.^47^ It should be remembered that programs of cognitive rehabilitation have been proven to be effective.^48^ These findings, combined with high prevalence rates of depression and cognitive impairment, support the need for programs that include interventions to treat both stroke complications, particularly those evaluating the prognostic usefulness and efficacy of treatment over the long-term.^21^

From a clinical perspective, knowing which patients are at increased risk of developing PSD associated with cognitive impairment may ameliorate prevention, detection, and early treatment of both conditions, consequently reducing their negative impact on recovery. Both depression and cognitive impairment are associated with worse stroke outcomes.^23,27,49,50^ In particular, impairment of executive function is a robust predictor of poor functional recovery after stroke.^27,29^ The depression-executive dysfunction syndrome is a stronger predictor of poor long-term survival than depression itself. Patients with both depression and executive dysfunction have shorter median survival than patients with neither depression nor executive dysfunction (6.6 versus 10.3 years).^51^

In conclusion, the present review suggests that the association between depression and cognitive impairment in the first three months after stroke is frequent and often underdiagnosed. Based on the current state of knowledge on the association between depression and cognitive impairment after stroke, we propose the following plan of action:

(a) Implementation of programs to increase diagnosis and treatment of PSD by non-psychiatrist physicians;
(b) Monitoring and evaluation of programs that include identification and interventions related to cognitive impairment;
(c) Definition of executive dysfunction as a possible element of a depressive syndrome or a predictor of PSD.
